# Supplementary material for: A fault-intrusion-tolerant system and deadline-aware algorithm for scheduling scientific workflow in the cloud
Source: PeerJ Comput Sci. 2021 Nov 2;7:e747. doi: 10.7717/peerj-cs.747 (PMC8576554; doi:10.7717/peerj-cs.747)
Supplement: Supplemental Information 6 [file peerj-cs-07-747-s006.zip › Myproject1.1/docs/org/cloudbus/cloudsim/class-use/Datacenter.html]

Uses of Class org.cloudbus.cloudsim.Datacenter


JavaScript is disabled on your browser.


- Overview
- Package
- Class
- Use
- Tree
- Deprecated
- Index
- Help

- Prev
- Next

- Frames
- No Frames

- All Classes

## Uses of Class org.cloudbus.cloudsim.Datacenter

- Packages that use Datacenter

  | Package | Description |
  | --- | --- |
  |  |  |
  | --- | --- |
  | org.cloudbus.cloudsim |  |
  | org.cloudbus.cloudsim.examples.power |  |
  | org.cloudbus.cloudsim.network.datacenter |  |
  | org.cloudbus.cloudsim.power |  |
  | org.workflowsim |  |
- - ### Uses of Datacenter in org.cloudbus.cloudsim

    Methods in org.cloudbus.cloudsim that return Datacenter

    | Modifier and Type | Method and Description |
    | --- | --- |
    |  |  |
    | --- | --- |
    | `Datacenter` | Host.`getDatacenter()` Gets the data center. |

    Methods in org.cloudbus.cloudsim with parameters of type Datacenter

    | Modifier and Type | Method and Description |
    | --- | --- |
    |  |  |
    | --- | --- |
    | `void` | Host.`setDatacenter(Datacenter datacenter)` Sets the data center. |
  - ### Uses of Datacenter in org.cloudbus.cloudsim.examples.power

    Methods in org.cloudbus.cloudsim.examples.power that return Datacenter

    | Modifier and Type | Method and Description |
    | --- | --- |
    |  |  |
    | --- | --- |
    | `static Datacenter` | Helper.`createDatacenter(java.lang.String name, java.lang.Class<? extends Datacenter> datacenterClass, java.util.List<PowerHost> hostList, VmAllocationPolicy vmAllocationPolicy)` Creates the datacenter. |

    Method parameters in org.cloudbus.cloudsim.examples.power with type arguments of type Datacenter

    | Modifier and Type | Method and Description |
    | --- | --- |
    |  |  |
    | --- | --- |
    | `static Datacenter` | Helper.`createDatacenter(java.lang.String name, java.lang.Class<? extends Datacenter> datacenterClass, java.util.List<PowerHost> hostList, VmAllocationPolicy vmAllocationPolicy)` Creates the datacenter. |
  - ### Uses of Datacenter in org.cloudbus.cloudsim.network.datacenter

    Subclasses of Datacenter in org.cloudbus.cloudsim.network.datacenter

    | Modifier and Type | Class and Description |
    | --- | --- |
    |  |  |
    | --- | --- |
    | `class` | `NetworkDatacenter` NetworkDatacenter class is a Datacenter whose hostList are virtualized and networked. |
  - ### Uses of Datacenter in org.cloudbus.cloudsim.power

    Subclasses of Datacenter in org.cloudbus.cloudsim.power

    | Modifier and Type | Class and Description |
    | --- | --- |
    |  |  |
    | --- | --- |
    | `class` | `PowerDatacenter` PowerDatacenter is a class that enables simulation of power-aware data centers. |
    | `class` | `PowerDatacenterNonPowerAware` PowerDatacenterNonPowerAware is a class that represents a non-power aware data center in the context of power-aware simulations. |
  - ### Uses of Datacenter in org.workflowsim

    Subclasses of Datacenter in org.workflowsim

    | Modifier and Type | Class and Description |
    | --- | --- |
    |  |  |
    | --- | --- |
    | `class` | `DatacenterExtended` DatacenterExtended extends Datacenter so as we can use CondorVM and other components |

- Overview
- Package
- Class
- Use
- Tree
- Deprecated
- Index
- Help

- Prev
- Next

- Frames
- No Frames

- All Classes
